# Rheological Characterization of Asphalt Fine Aggregate Matrix Using Dynamic Shear Rheometer

**DOI:** 10.3390/polym11081273

**Published:** 2019-07-31

**Authors:** Xiangbing Gong, Zejiao Dong, Haipeng Wang, Xianyong Ma, Huanan Yu, Kaikai Hu

**Affiliations:** 1 State Engineering Laboratory of Highway Maintenance Technology, Changsha University of Science and Technology, Changsha 410114, China; 2 School of Transportation Science and Engineering, Harbin Institute of Technology, Harbin 150090, China; 3 Sichuan Highway Planning, Survey, Design and Research Institute Limited Ltd., Chengdu 611130, China

**Keywords:** rheology, dynamic shear rheometer, industrial computed tomography, fine aggregate matrix

## Abstract

Asphalt fine aggregate matrix (FAM) is a predominant component directly related to field performances of hot asphalt mix (HMA), it is necessary to investigate material properties of FAM. Prior to preparing FAM specimens, the asphalt content was calculated by keeping the filler–bitumen (*FB*) ratio the same as in the corresponding HMA. A non-destructive fabrication method instead of coring and cutting methods was developed to compact FAM cylinders, and the joint base was designed to be concentric with the loading axis of testing system. Rheological responses of FAM were studied using the dynamic shear rheometer (DSR). Two repeated tests prove that the FAM compactor and the jointed base meet the requirement of data validation. Results show that rheological performances of FAM are significantly affected by asphalt content, gradation, air void content, and testing frequency. Air void is concluded to be the decisive factor which influences the stability of FAM, and the fiber is demonstrated to play a role on enhancing the flow resistance of FAM-F even though with the richest asphalt content.

## 1. Introduction

**Figure 1:**
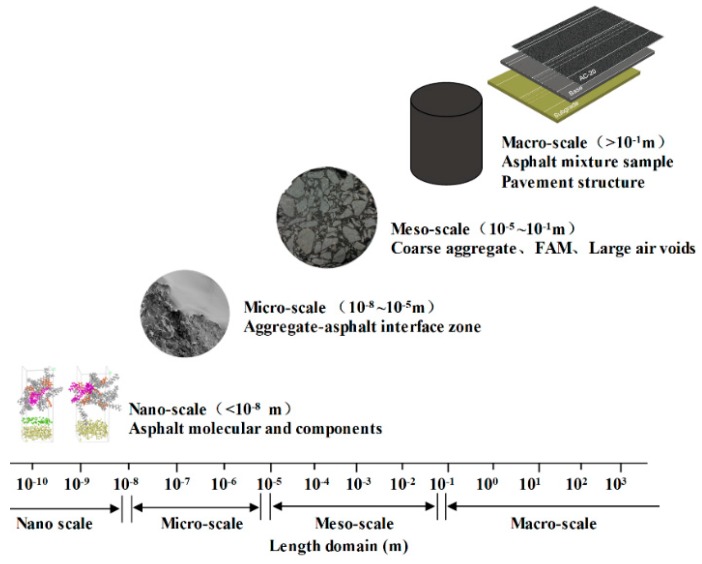
Scale classification of HMA: nano-scale, micro-scale, meso-scale and macro-scale.

Due to undoubtable advantages—such as lower noise, quick construction, easy maintenance, and comfortable driving—asphalt mixture is the primary material form adopted in pavement construction. In current pavement design methods, asphalt mixture is treated as a homogeneous substance. This assumption is mainly employed to simplify the procedure of structural design of pavement. However, with the development of mechanical technique, the multi-scale has been characterized aiming at comprehensively understanding on fundamental construction materials subjected to various loads and temperatures. Both theory [1] and numerical methods [2,3] proved that the composite like asphalt mixture was the typically scale-dependent material (Figure 1). As a universal asphalt mixture type, hot mix asphalt (HMA) is generally divided into four parts which are large air void, coarse-aggregate–asphalt interface zone, asphalt fine aggregate matrix (FAM), and coarse aggregate, respectively. Based on scale classification shown in Figure 1 for HMA, FAM consists of asphalt binder, fine aggregate, filler, and small air void [4]. Additionally, FAM is the main component directly related to distresses existing in real asphalt pavements. The characteristic length of high-temperature flow displacement (rutting) and low-temperature or fatigue crack growth (cracking) of asphalt pavement is seemed to be sensitive to the FAM properties [4]. Therefore, it is essential to evaluate FAM performances and establish a relationship between FAM and HMA.

One prior research interest was focused on the property characterization of FAM. Dynamic testing was widely adopted to characterize dynamic properties of binders [5,6] and asphalt mixtures [7,8]. In addition, researchers developed similar experiments to conduct dynamic tests for FAM, and extensive results were summarized as below. The viscoelastic and tensile properties of FAM were sensitive to the volumetric factor, and precise components of FAM should be carefully considered [8,9]. Cylindrical FAM samples were cored to be included in gravimetric sorption experiments, and type of aggregate and asphalt binder were decisive factors to affect the moisture distribution in FAM [10]. Dynamical mechanical analyzer (DMA) was used to test the fatigue of FAM and a fatigue damage parameter was summarized eventually [11]. As basic properties, material parameters of FAM were utilized in numerical analysis at multiple scales [12,13,14]. Although prior studies aimed at the rheological characterization of FAM, the air void size distribution of FAM was ignored.

Material design and preparation of FAM was the second main research interest which focused on obtaining appropriated material components. It is obvious that asphalt binder should be absorbed by aggregate, hence the asphalt content of FAM could be calculated by excluding the asphalt absorbed by coarse aggregate to form the interface zone [15]. Based on definitions in a handbook [16], the effective asphalt content was provided to achieve this goal. The next step was to fabricate specimens, a conventional method was to compact FAM cylinder by using the Superpave gyration compactor (SGC) [15,17]. The rich binder of FAM leads to displacements at high temperatures because of its large weight. Consequently, a small cylinder cored from cylindrical samples not only reduces its mass but also be assembled into clamps of DSR and DMA [9,11]. One issue is concluded as that cutting and coring are destructive processes which might generate micro defects in FAM samples. Another one is known to be the asphalt leak at a high temperature from the gap of the mold because of the rich binder content of FAM while using SGC compactor due to its high compress stress. Therefore, it is necessary to develop a nondestructive and stable material preparation method to fabricate FAM. 

The first objective is to evaluate rheological performances of FAM including the effect of gradation and small air void. Six types of FAM were obtained based on the corresponding hot-mix asphalt (HMA) mixtures. Frequency sweep test, fatigue test, relaxation test, and static creep test of FAM were conducted via a DSR named as DHR-II (Discovery Hybrid Rheometer, TA, New Castle, DE, USA). The X-ray industrial computer tomography (ICT, Phenix, Berlin, Germany) was used to determine the air void distribution in FAM at a high resolution [18]. The second objective is to develop a non-destructive specimen fabrication method. Instead of cutting test, coring test, and high press compaction, a scaled-down FAM compactor was designed to compact small FAM cylinders by keeping the same similarity of size and compaction energy as them in the HMA Marshall compaction [19]. A joint base was designed to be a connection between FAM samples and DHR clamps. Thus, a couple of developed clamps were created to achieve the objectives of this study.

## 2. FAM Material Design

**Figure 2:**
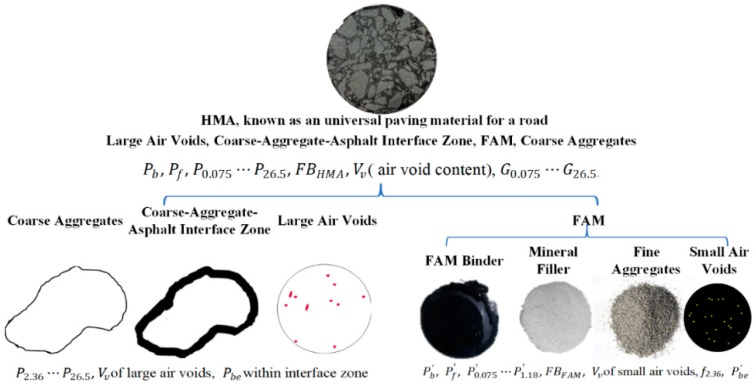
Schematic image to calculate asphalt–aggregate ratio in FAM.

**Figure 3:**
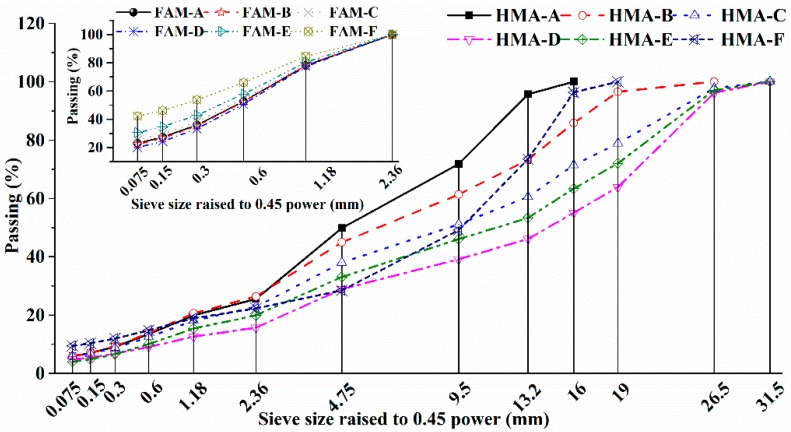
Gradation curves for six types of HMA and the corresponding FAM.

FAM is a major component of HMA at meso-scale known to exclude coarse aggregate as well as the absorbed asphalt by coarse-aggregate–asphalt interface zone, large air void (Figure 2). The nominal maximum aggregate size (NMAS, mm) of FAM was set to be 1.18mm. Material components’ content and gravity of Six HMAs were tested. The ratio among filler (sieve size smaller than 0.075 mm, $P_{f}^{\prime }$) and fine aggregate ($P_{n}^{\prime }$, *n* means sieve size) ($P_{f}^{\prime }:P_{0.075}^{\prime }:P_{0.15}^{\prime }:P_{0.3}^{\prime }:P_{0.6}^{\prime }:\;P_{1.18}^{\prime })$ was keep the same as it in HMA ($P_{f}:P_{0.075}:P_{0.3}:P_{0.6}:P_{1.18}$) and the total percentage of filler and fine aggregate in FAM was 100. When gradation curves of six types of HMA were given, and their corresponding FAM gradations could be achieved finally. Except for a gap gradation (HMA-F), Figure 3 implies that another five mixtures are dense-graded. HMA-D and HMA-E are used as base mixtures, others are applied as surface or intermediate layers. FAM-F is the finest gradation followed by FAM-E, and others are similar. In addition, FAM-C is the finest and FAM-D has more large size of aggregate than other four gradations. Filler, aggregates and fibers were from a local quarry in Changchun, China. Binder was from Shandong Jianzhu University in Jinan, China.

After the determination of gradation, the predominant procedure for material design is to calculate the asphalt content in FAM. As mentioned above, one part of asphalt binder in HMA is absorbed by coarse aggregate, and another part is known as the effective asphalt existing in FAM. It is essential to achieve the effective asphalt content which refers to the free binder without absorbing by aggregate [16]. 

Firstly, the bulk specific gravity (saturated surface dry) for coarse and fine aggregate with individual size was obtained already [20,21]. Then the bulk specific gravity for total aggregate can be calculated by Formula (1)

(1) $$G_{sb}=\left(P_{f}+P_{0.075}+\ldots +P_{n}\right)/\left[\left(P_{f}/G_{f}\right)+\left(P_{0.075}/G_{0.075}\right)+\ldots +\left(P_{n}/G_{n}\right)\right]$$

where

*G_sb_* = bulk specific gravity for the total aggregate of HMA;
*P_n_* = individual percentages by weight of aggregate and filler in HMA; and
*G_n_* = individual bulk specific gravity of aggregate and filler in HMA.

Secondly, effective specific gravity of aggregate can be determined based on the maximum specific gravity of paving mixture defined in a specification [22]. This gravity includes the volume of all air void in aggregate except the spaces for the absorbed asphalt, it can be determined using Formula (2)

(2) $$G_{se}=\left(P_{mm}-P_{b}\right)/\left(P_{mm}/G_{mm}-P_{b}/G_{b}\right)$$

where

*G_se_* = effective specific gravity of aggregate of HMA;
*G_mm_* = maximum specific gravity of paving mixture of HMA;
*P_mm_* = percent by weight of total losse mixture of HMA, the value is 100;
*P_b_* = asphalt content in HMA, percent by total weight of HMA; and
*G_b_* = specific gravity of asphalt, it is 1.09 for polymer modified asphalt.

Third, the percentage of asphalt absorption can be achieved and the effective asphalt content can be calculated by Formulas (3) and (4)

(3) $$P_{ba}=100\left(G_{se}-G_{sb}\right)G_{b}/G_{se}G_{sb}$$

(4) $$P_{be}=P_{b}-P_{ba}P_{s}/100$$

where

*P_ba_* = absorbed asphalt by all aggregate in HMA, percent by weight of aggregate;
*P_be_* = effective asphalt content in HMA, percent by total weight of HMA; and
*P_s_* = aggregate content in HMA, percent by total weight of HMA.

The filler–bitumen ratio (*FB*) is an important index which shows a significant impact on the performances of HMA [23], and the range of *FB* must be appropriate in the paving mixture. *FB_HMA_* is defined as the ratio of filler (*P_f_*) to the effective asphalt (*P_be_*) by weight seen in Formula (5). Therefore, the asphalt content in FAM is calculated by keeping *FB* the same as it in HMA. Ultimately, $P_{b}^{\prime }$ can be deduced in Formula (8) by solving Formulas (6) and (7) combined with the *FB_HMA_* calculated in a given HMA gradation

(5) $$FB_{HMA}=P_{f}/(P_{b}-P_{ba}P_{s}/100)$$

(6) $$FB_{FAM}=P_{f}^{\prime }/P_{be}^{\prime }$$

(7) $$P_{be}^{\prime }=P_{b}^{\prime }-P_{ba}^{\prime }\left(100-P_{b}^{\prime }\right)/100=P_{b}^{\prime }-P_{ba}f_{2.36}\left(100-P_{b}^{\prime }\right)/100$$

(8) $$P_{b}^{\prime }=\left(100P_{f}^{\prime }/FB_{HMA}+100P_{ba}f_{2.36})/(P_{ba}f_{2.36}+100\right)$$

where

$P_{b}^{\prime }$ = asphalt content in FAM, percent by total weight of aggregate;
$P_{f}^{\prime }$ = filler content in FAM, percent by weight of aggregate;
$P_{ba}^{\prime }$ = absorbed asphalt by FAM in HMA, percent by weight of aggregate;
$P_{be}^{\prime }$ = effective asphalt content in FAM, percent by total weight of FAM;
*f*_2.36_ = 2.36 mm sieve passing rate in HMA gradations, percent by weight of aggregate;
*FB_HMA_* = ratio of filler to effective asphalt content in HMA; and
*FB_FAM_* = ratio of filler to effective asphalt content in FAM.

Former studies from our team has used the same method to calculate the asphalt–aggregate ratio in FAM [24,25]. Results of parameters mentioned preciously are shown in Table 1. Obviously, finer gradation contributes to richer asphalt content in FAM. The asphalt content in FAM-F is the richest one, followed by FMA-E. FAM-A, FAM-C, and FAM-D show similar results, although FAM-D is known to be the coarsest gradation and the smallest *FB_FAM_* increases the asphalt content. This observation presents that the poorest asphalt content is found in FAM-B.

## 3. Experiments

### 3.1. Speciment Preparation Test

**Figure 4:**
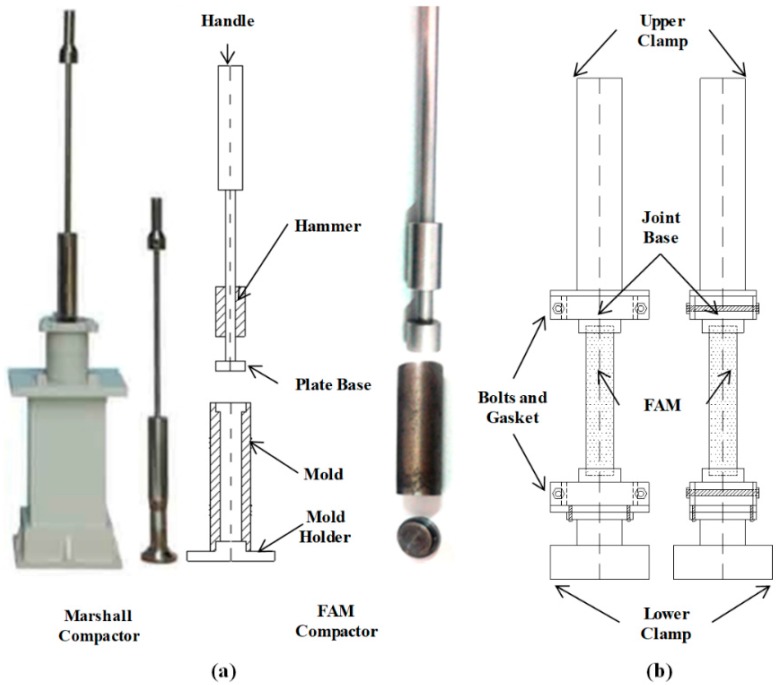
Compactors and DHR clamps: (**a**) Marshall compactor and FAM compactor, and (**b**) FAM cylinder, metal base, and rectangle clamps.

In this study, a non-destructive preparation method was provided to solve the cutting and coring issue. In accordance with the specification used to prepare Marshall testing specimens [19], a FAM compactor was developed to prepare small FAM cylinders base on a scale-down factor. Instead of SGC compactor, the main advantage of falling hit compaction is the hit press is appropriate to avoid asphalt leak while compacting FAM. In Figure 4a, the FAM compactor consists of five parts made of metal: a handle to be holed by hands, a falling hammer, a place base to be hit by the hammer, a mold, a mold holder. A 12 mm diameter cylinder with 45 mm height is the specimen geometry for FAM, but a 101.6 mm diameter cylinder with 63.5 mm height is adopted in the Marshall test (Figure 4a). Additionally, the Marshall compaction hammer (Figure 4a) has a 4536 g sliding weight and a free fall of 457.2 mm. Based on the similarity analysis, the scale-down factor (0.71) is referred as to the ratio of height of FAM samples to Marshall samples. Therefore, the free-falling height for the FAM hammer is 324.0 mm. Another essential issue for the FAM compactor is the mass of hammer which is calculated by remaining the same compaction energy per volume unit (J/cm^3^) as it in the Marshall test. Ultimately, the mass of FAM compaction hammer is set to be 63.3 g.

The next step was to fabricate FAM specimens by compacting through the FAM compactor, and then FAM samples can be installed into the rectangle clamps of DHR connected with a joint base shown in Figure 4b. These steps are recommended for preparing FAM specimens: (a) Prepare raw materials such as fine aggregate, SBS (styrene–butadiene block copolymer) polymer modified asphalt, and mineral filler. Fine aggregate and filler content can be seen in Figure 3, asphalt content can be seen in Table 1. All raw materials’ physical properties match the specification limits adopted in China. (b) After four hours’ heating in an oven, fine aggregate, SBS polymer modified asphalt, and mineral filler were blended together at an appropriate temperature, the blending duration increases to 5 min because of the rich asphalt content. (c) After the blending, a certain amount of FAM was weighted using a balance sensitive to 0.01 g, and then the FAM batch was immediately put into the oven to avoid the decrease of temperature. (d) Remove the FAM compactor from the oven and spray the lubricant onto the metal surfaces contacted with FAM, then the FAM batch reheating for 5 min in the oven was immediately dumped into the compaction mold placed in the mold holder on a smooth and level surface. (e) Spade the FAM and smooth the surface to a slightly rounded shape, place the plate base assembly on the mold and hold the handle to make the hammer nearly perpendicular to the mold holder, then apply 75 blows with the compaction hammer using a free fall of 324.0 mm. (f) Remove the plate base and put the mold, holder and FAM into the oven for another 5 min reheating, then reverse and assembly the mold, apply another 75 blows to the other surface of the reversed specimen. (g) After the compaction, allow the specimen to cool in air to an extraction temperature, and then assembly the plate base to remove the FAM cylinder from the mold by pushing the handle with hands. After the extraction of FAM cylinders, the specimens were placed on a smooth, level surface to cool overnight.

### 3.2. Propperty Investigation Tests

**Figure 5:**
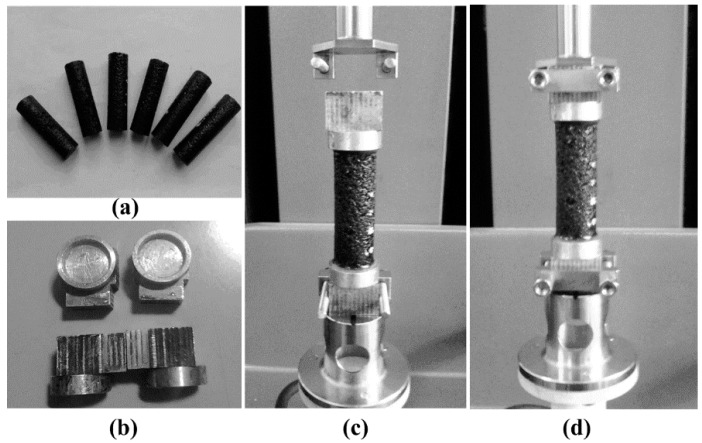
FAM cylinders and set-up: (**a**) six types of FAM cylinder, (**b**) joint bases, (**c**) one base with fixed FAM installed into the lower clamp, and (**d**) the other base installed into the upper clamp.

In this study, FAM samples (Figure 5a) were fabricated using the FAM compactor shown in Figure 4a. Prior to testing the sample, the connection between FAM cylinders and clamps of DHR needs to be accomplished. To assembly FAM cylinders into the rectangle clamps of DHR, the joint base (Figure 5b) was designed to be embed into the rectangle clamps using bolts and gaskets (Figure 4b). The top part of joint base is a cylindrical notch and the bottom part is a base which can be totally plugged into the DHR clamps. To avoid eccentric force, the top notch of joint base, lower clamp, and upper clump must be concentric after set-up. Therefore, the joint base was fabricated to be concentric with the clamp by keeping it fixed into the clamp while conducting machining processes. The super epoxy was adopted to fix cylinders into the notch (*Φ*12 × *h*2.5 mm), and the curing duration was set to be 24 h by placing FAM cylinder and two joint bases on a smooth and level surface. The next step was to install the joint base into the clamp. One base was embedded into the lower clamp of DHR first (Figure 5c), and then move down the upper clamp slowly until another base was completely locked into the upper clamp (Figure 5d). Ultimately, bolts and gaskets were fastened to hardly touch the joint bases preventing from any movement.

After the experimental set-up, DHR-II was adopted to conduct mechanical characterization of FAM [26]. The main advantage of DHR-II is the precise strain detection and stable gap measurements even for a solid sample like FAM. To assure that the temperature of FAM reaches the target value, the soak duration in the environmental chamber is set to be 30 min. Another essential factor is that all tests were conducted under the limitation of LVE (linear viscoelasticity). The detailed description of five tests is shown as below:

(a) ICT scanning test: ICT was used to explore the internal air void in FAM; there were four samples for each FAM group. The voxel resolution was set to be 69.09 μm, scanning voltage was 190 kV and current was 100 μA, each sample turned a circle with 1000 scanning images. (b) Frequency sweep test: seven temperatures were included (−5 °C, 0 °C, 15 °C, 30 °C, 45 °C, 60 °C, 75 °C), the frequency changed from 0.1 Hz to 30 Hz, all tests were kept in the stress-controlled mode. (c) Fatigue test: all FAM groups were subjected to a stress-controlled fatigue test at 25 °C, the shearing stress was set to be 100 kPa, the duration was 4000 s, the frequency was 10 Hz. (d) Relaxation test: a constant shear strain of 0.01% was applied to obtain the relaxation modulus of FAM cylinders at 0 °C, the duration lasted around 3600 s. (e) Static creep test: a constant shear stress of 3000 Pa was applied to test the creep strain of FAM cylinders at 45 °C, the duration lasted 3600 s. 

**Figure 6:**
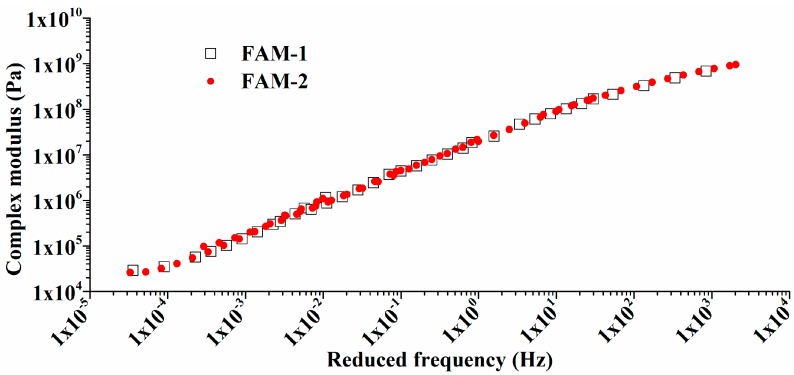
Repeatability verification: master curves of |G^*^| for two different tests at 30 °C.

Repeatability is a fundamental factor that needs to be proven for testing FAM, two FAM cylinders with the same gradation were subjected to several frequency sweep tests. Master curves of |G^*^| (complexed modulus) at 30 °C were obtained by different operators. Two curves in Figure 6 show that there is almost no difference between two groups. Consequently, DHR-II is demonstrated to be feasible to test FAM cylinders compacted by the FAM compactor and tested by rectangle clamps. 

## 4. Results and Discussion

### 4.1. ICT Scanning Test

**Figure 7:**
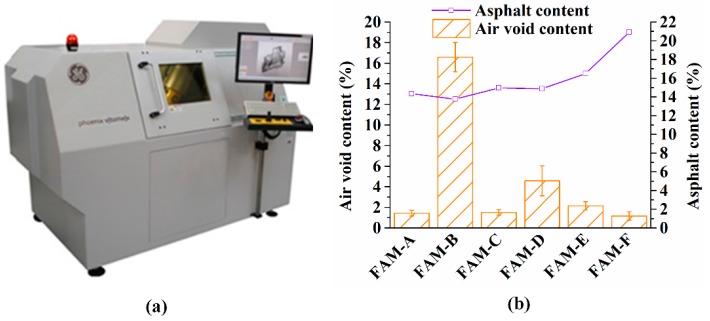
Air void in FAM: (**a**) Phoenix v|tome|xs ICT with micro focus, and (**b**) the air void content and the asphalt content in FAM.

**Figure 8:**
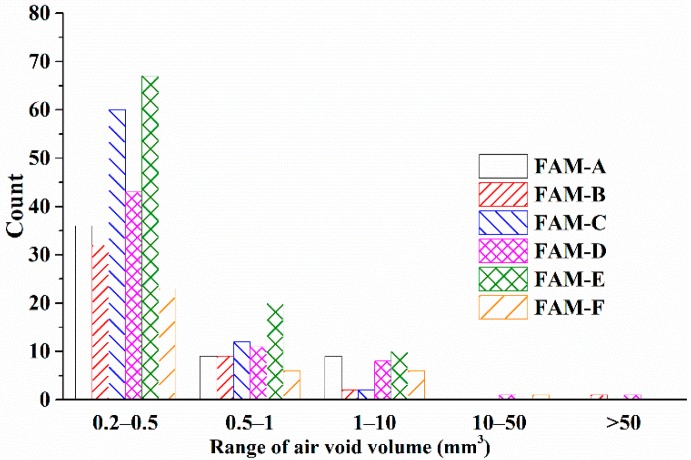
Volume distribution of air voids in FAM.

The air void content of bituminous materials is an essential factor to control quality of HMA as well as FAM. Known as a non-destructive tool, ICT has been utilized to explore the volumetric feather of asphalt mixture [27]. Before scanning samples, ICT (Figure 7a) detected the X-ray background information only penetrating air without any sample. Therefore, the detector could recognize the air void by comparing with the background signal. The open and close air void were detected effectively by distinguishing the grayscale via software of *VGStudio MAX*. The relationship between the asphalt content and the air void content is shown in Figure 7b. The air void content of FAM is significantly affected by the asphalt content. The greatest air void content (16.60%) is seen in FAM-B which has the poorest asphalt content (13.77%). The smallest air void content is found in FAM-F which has the richest asphalt content (20.92%). On the other hand, the gradation is another decisive factor to influence the air void content in FAM. Although the asphalt content in FAM-D is only 0.09% smaller than in FAM-C, the air void content in FAM-D is 3.07% greater than in FAM-C. Because the individual percentages of fine aggregate in FAM-D are greater than in FAM-C except for the filler content. The difference of the air void content between FAM-A and FAM-C is only 0.06%, which can be demonstrated by a finding that the asphalt content and the gradation of fine aggregate are similar between FAM-A and FAM-C. Additionally, Figure 8 shows that the air void size is small and dependent on the FAM type. The air void volume indicates the size distribution of air void in FAM. Figure 8 exhibits that the air void in FAM-B are larger than them in other FAM samples. The largest air void volume is 649.1 mm^3^ in FAM-B, followed by 116.9 mm^3^ seen in FAM-D, and the third largest one can be found in FAM-F (43.9 mm^3^). The number of air voids in FAM-F is the least, but the large size is the primary type of air void, which shows a good relationship to big air void size in SMA mixtures [28]. The size in FAM-A, FAM-C, and FAM-E is proved to be smaller based on Figure 8. It can be concluded that more asphalt results in more small air voids in FAM except FAM-F. 

### 4.2. Frequency Sweep Test

**Figure 9:**
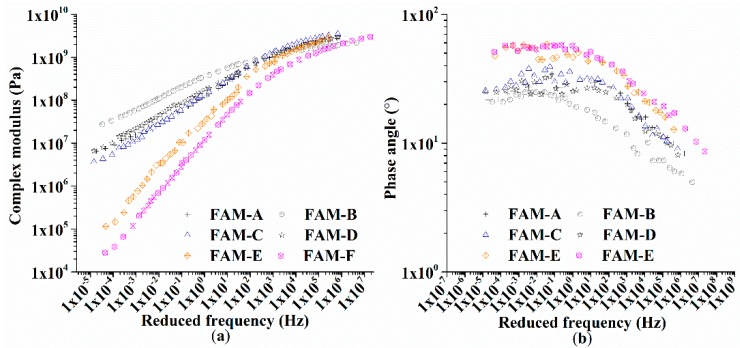
Master curves of FAM at 30 °C: (**a**) complex modulus (|G^*^|), and (**b**) phase angle (°).

**Figure 10:**
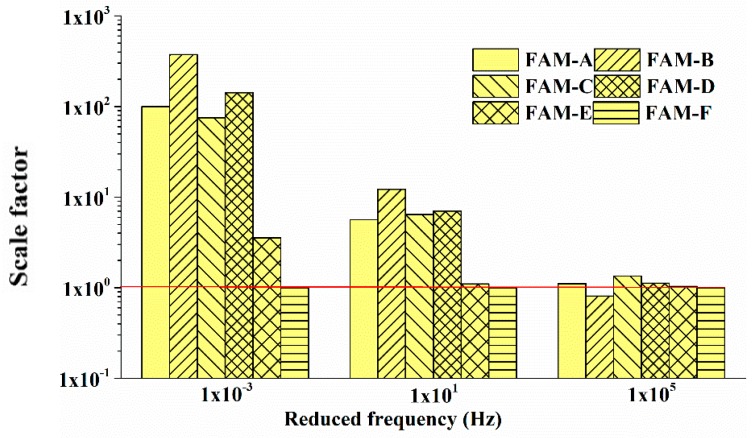
Scale factor of complex modulus at three kinds of frequency.

Base on the time-temperature superposition principle [29], master curves of FAM at 30 °C were determined by shift factors at other temperatures. Master curves of the complex modulus (|G^*^|) and the phase angle (°) are shown in Figure 9. It is obvious that the |G^*^| shows significant changes caused by asphalt content, air voids, and frequency. Figure 9 exhibits that increase in the asphalt content reduces the modulus value and raises the phase angle obviously [9]. The asphalt content is demonstrated to be the most predominant factor to affect the frequency sweep test of FAM. The |G^*^| curves of FAM-E and FAM-F shown in Figure 9a are found to have the similar tendency as well as the curves of FAM-A, FAM-C, and FAM-D. Additionally, these two contrasting groups show similar phase angle curves in Figure 9b. It can be explained by the fact that these two groups content similar values of asphalt content. Therefore, the asphalt content adopted in real asphalt pavements needs to be carefully considered to generate a strong FAM material. The poorest asphalt content and the largest air void size contributes to the increase of the elastic ratio in FAM-B, because FAM-B presents a flat modulus curve and the smallest phase angle. In Figure 7a, the |G^*^| gaps among all six types of FAM are influenced by the frequency. A scale factor shown in Figure 10 indicates fluctuations shown in Figure 9a. FAM-F was selected to be the reference group as a number of one, the scale factor of other FAM was obtained based on diving by the |G^*^| of FAM-F at three kinds of frequency (10^−3^ Hz, 10^1^ Hz, 10^5^ Hz). 

The red line in Figure 10 represents the scale factor of FAM-F which is set to be one. It can be seen that the modulus values for all groups are similar at a high frequency at 30 °C, because all values of scale factor are around one. However, the scale factor for FAM-B changes from 0.81 at 10^5^ Hz to 375.68 at 10^−3^ Hz. It is obvious that the modulus gaps increase obviously with the decrease of frequency. Consequently, the mechanical responses of FAM can be easily differentiated undergoing a long-lasting heavy traffic or a high temperature. Moreover, the traffic and the temperature must be included into a FAM design approach for future filed application. By comparing the scale factor between FAM-E and FAM-F or FAM-A, FAM-B, and FAM-C, it is found that these two groups almost behave the same. 

### 4.3. Fatigue Test

**Figure 11:**
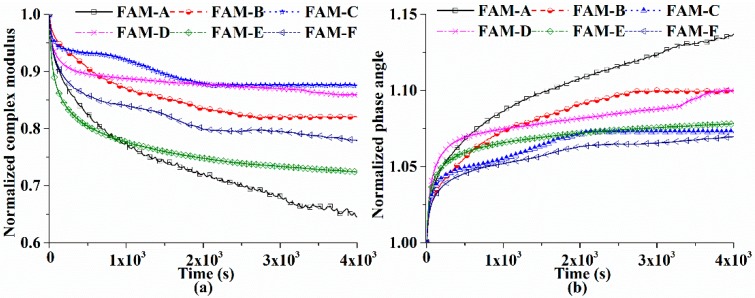
Normalized curves for fatigue tests at 25 °C: (**a**) the normalized complex modulus ((|G^*^|), and (**b**) the normalized phase angle (°).

A stress-controlled fatigue test was conducted to explore the influence of repeated loads in asphalt pavements [30]. To investigate the effect of fatigue, normalized complex modulus (Figure 11a) and normalized phase angle (Figure 11b) were calculated to differentiate the resistance to fatigue of FAM. Figure 9b presents that the ratio of storage modulus for FAM decreases caused by the fatigue along with the increase of phase angle. 

Fatigue would degrade the recoverable capability of FAM. Figure 11 exhibits that FAM-C has the smallest decrease percent of modulus at the end of the test, and followed by FAM-D. One obvious difference between FAM-C and FAM-D is that FAM-D reaches a stable condition at the beginning, but the modulus of FAM-D goes down significantly at the end. It is concluded based on the rapid growth of normalized phase angel in Figure 11b for FAM-D at the end of fatigue test. Therefore, the gradation of FAM is testified to influence fatigue resistance subjected to moving vehicles on the pavements. FAM-B shows better fatigue resistance than FAM-A with a rapid decrease of complex modulus, and the growing phase angle in FAM-A presents that fatigue reduces the elastic proportion. Because FAM-A and FAM-B have similar gradation, the differences illustrate that sufficient air voids enhance the capability to bear repeated vehicle loads. FAM-E and FAM-F show poor resistance to fatigue, but the percent decrease of storage modulus is found to be smaller. A study concludes that SMA mixtures are not as strong as AC mixtures when it comes to fatigue, and more asphalt in SMA mixtures means more fatigue damage [31]. Therefore, richer asphalt content and finer gradation in FAM shorten the fatigue life. Based on the discussion mentioned above, the fiber in FAM-F is proven to make the fatigue life of FAM-F greater than FAM-E.

### 4.4. Relaxation Test

**Figure 12:**
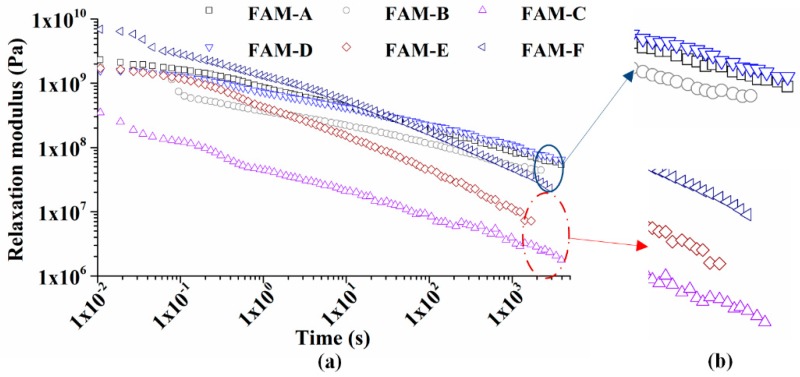
Relaxation modulus versus time of FAM at 0 °C: (**a**) whole image, and (**b**) enlarged image.

Thermal crack is a common distress happening in asphalt pavements in cold regions due to the limited relaxation rate, and the crack is observed to usually occur within FAM part in HMA. In Figure 12a, the initial relaxation modulus of FAM ranges from 1.8 × 10^9^ Pa to 6.9 × 10^9^ Pa, because a low temperature is equivalent to a high frequency seen to reduce the differences of modulus in Figure 9a. The slope of logarithmic relaxation modulus versus logarithmic time curve indicates the rate at which FAM releases thermal stresses caused by shrinkage.

The asphalt content exhibits a significant impact on the slope. More asphalt in FAM contributes to the better relaxation resistance, since FAM-E and FAM-F show a rapider decline of relaxation modulus than others. The relaxation rate of FAM-C is remarked as the third highest, which could be explained by a finding that FAM-C has richer asphalt and finer gradation than FAM-A, FAM-B, and FAM-D. Therefore, FAM-C, FAM-E, and FAM-F are less likely to crack in cold climates. However, other FAMs are easier to crack caused by the smaller stress releasing rate. At the end of relaxation test, the trend of modulus curves indicates the relaxation rate of FAM subjected to an extremely cold climate. In Figure 12b, details present that the curves of FAM-A, FAM-B, and FAM-D tend to be flat especially for FMA-B, which implies that thermal stresses are difficult to be relaxed. The slope of FAM-C, FAM-E, and FAM-F is seen to be not affected by the time. Consequently, insufficient asphalt is a main factor to degrade the low temperature properties of FAM, especially when suffering a long-lasting cold climate.

### 4.5. Static Creep Test

**Figure 13:**
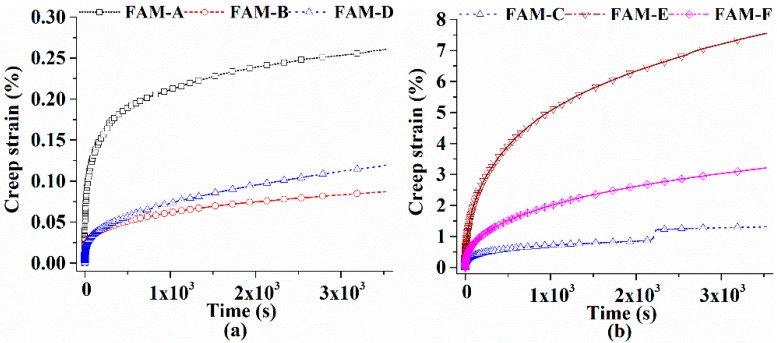
Creep strain versus time of FAM at 45 °C: (**a**) small strain group, and (**b**) great strain group.

Figure 13 tells that the creep strain at the end of creep test for all FAM ranges from 0.087% to 7.552%, this large changing range can be explained by the scale factor at 10^−5^ Hz in Figure 10. To clearly illustrate the differences of creep strain, two FAM groups are named as small strain group and large strain group. It is reasonable that larger modulus at high temperature or low frequency means smaller creep strain. The scale factor at 10^−5^ Hz shows good predictions to the creep strain values except for FAM-F, because FAM-E other than FAM-F has the largest strain and the rapidest growth, even the scale factor of FAM-E is greater than 1. Rich asphalt content in FAM is a disadvantage to degrade the creep resistance. Namely, FAM-F is supposed to be the poorest sample accounting for the creep test. The fiber adopted in FAM-F may enhance the asphalt–aggregate bonding, which reduces the effect of easily flowing asphalt at high temperature. This finding proves that not only the skeleton of coarse aggregate but the stable FAM improves the high temperature performance of SMA mixture. In Figure 13b, FAM-C shows a jumping strain increment and then keeps stable. Denser gradation and less air void in FAM-C are anticipated to lead to the instability. A sufficient amount of air void in FAM is seemed to provide spaces allowing binder flow under a long-term traffic and avoid the instability. 

## 5. Conclusions

To avoid destructive fabrication, a novel FAM compactor was developed by keeping parameters similar as them in the Marshall test. Rheological properties of FAM were investigated using the DSR (DHR-II, TA). ICT scanning test was used to detect the air void at a precise resolution, frequency sweep test, fatigue test, relaxation test, and static creep test were conducted to distinguish FAM properties. Based on results mentioned preciously, conclusions are summarized as below. 

FAM compactor is feasible to fabricate FAM samples due to non-destructive processing and avoiding the flow asphalt leak at high temperature. The set-up and the protocol based on the concentric joint base is proved to be effective by the repeatability test. 

Asphalt content, FAM gradation, and air void size are decisive factors to affect the complex modulus of FAM obtained by the frequency sweep test. The effect of these factors is obviously dependent on the frequency.

Rich asphalt content and dense gradation of FAM are adverse factors to degrade the fatigue resistance at intermediate temperature and the creep resistance at high temperature. While these two factors generate strong FAM against the thermal cracking at low temperature.

A sufficient amount of air void in FAM must be optimized while conducting material design, because it is the most predominant factor to affect the stability of FAM in HMA subjected to a long-term shear flow. Fibers and air void in FAM-F (SMA type) improve the asphalt–aggregate bonding property, and reduce the adverse effect of rich asphalt and fine gradation in FAM-F. This finding in FMA-F exhibits a similar characteristic to SMA type of HMA known for its stable service properties. 

## 6. Future Work

The research team will utilize numerical simulation tools to analyze mechanical characteristics of six HMAs as a mesoscopic composite by combining material parameters of six FAMs, coarse-aggregate–asphalt zone and large air void distribution. The next step is to clarify the accuracy of numerical models via comparing with experimental results of HAM such as the dynamic modulus test. The main goal of future works is to establish the relationship between simulated FAM and real FAM in HMA.

## Figures and Tables

**Table 1:** Parameters related to the determination of asphalt content in FAM (percentage)

HMA											*FB_FAM_*		FAM		
Name	Type	NMAS	*P_b_*	*G_sb_*	*G_se_*	*G_a_*	*P_ba_*	*P_be_*	*P*_2.36_	*P_s_*		Name	NMAS	*P′_f_*	*P′_b_*
HMA-A	AC *	13	4.58	2.607	2.643	1.09	0.56	4.05	25.58	95.42	1.39	FAM-A	1.18	23.07	14.33
HMA-B		20	4.49	2.609	2.642		0.53	3.99	26.36	95.51	1.41	FAM-B		22.38	13.77
HMA-C		25	4.31	2.608	2.641		0.52	3.81	22.78	95.69	1.46	FAM-C		25.46	14.96
HMA-D	ATB *	25	3.85	2.605	2.639		0.54	3.33	19.94	96.15	1.16	FAM-D		20.06	14.87
HMA-E		30	3.47	2.607	2.640		0.52	2.97	15.67	96.53	1.56	FAM-E		30.63	16.49
HMA-F	SMA *	16	6.10	2.595	2.635		0.64	5.51	22.29	93.90	1.60	FAM-F		42.17	20.92

* AC represents asphalt concrete, ATB means asphalt-treated base, and SMA is short for stone mastic asphalt.
